# Anti-pandemic resilience assessment for countries along the Belt and Road route

**DOI:** 10.3389/fpubh.2023.1152029

**Published:** 2023-11-02

**Authors:** Laijun Zhao, Mengmeng Min, Xiaoyan Huang, Ying Qian, Lixin Zhou, Pingle Yang

**Affiliations:** ^1^Business School, University of Shanghai for Science and Technology, Shanghai, China; ^2^Emergency Management Office, Shanghai Municipal Center for Disease Control and Prevention, Shanghai, China

**Keywords:** Belt and Road countries, COVID-19, pandemic response, anti-epidemic, combined methods, empowerment method, VIKOR method

## Abstract

**Background:**

The COVID-19 pandemic is sweeping the world, and countries along the Belt and Road (B&R) route have also been hit hard. However, the impact varied greatly from country to country, some severely and others mildly. What factors have led to such a wide variation?

**Method:**

In this paper, we considered institutional, infrastructural, economic, social, and technological resilience as components of overall anti-pandemic resilience, and constructed a set of indicators to evaluate this resilience for B&R countries in 2020. We evaluated the anti-pandemic resilience using the combined empowerment–VIKOR method, and classified the countries into different resilience levels by means of hierarchical clustering. The validity of the evaluation indicator system was verified by analyzing the consistency between the actual performance and the assessed resilience.

**Results:**

The ranking results showed that Israel and Bahrain were representative of countries that had the highest resilience, Hungary and Estonia represented countries with moderate resilience, and Laos and Cambodia represented countries with the lowest resilience. We also found that countries with high resilience had much better institutional and economic resilience than countries with moderate resilience, whereas countries with low resilience lagged behind in both infrastructural and social resilience. Based on these findings, policy recommendations were offered to help B&R countries respond to future pandemics.

## 1. Introduction

The Belt and Road Initiative, launched by China in 2013, is a vast development project that has substantially impacted the economies and societies of the countries situated along its routes. In addition to prioritizing economic collaboration and infrastructure construction, the Belt and Road Cooperation Initiative also directed attention to public health, implementing several cooperative endeavors. These initiatives aimed to narrow the healthcare infrastructure disparities in low-and middle-income nations and involved the establishment of global health research institutes in partnership with universities, with the objective of enhancing medical and healthcare standards (1).

However, the sudden global spread of COVID-19 in 2020 has impeded the progress of the initiative, which has served as a vital test of the effectiveness of the Belt and Road development and cooperation initiatives. Countries located along the Belt and Road have been particularly hard hit, with many of them being developing countries where controlling the pandemic and driving recovery may be more challenging (2). Nevertheless, certain countries have exhibited exceptional resilience and have successfully mitigated the pandemic’s impact, returning to normalcy. What factors have led to such a wide variation? The underlying reasons for such discrepancies require further examination. In this paper, we construct a set of indicators to evaluate the anti-pandemic resilience of B&R countries in 2020. This assessment can identify successful and underperforming countries, pinpoint areas for improvement and address weak links. The policy advice formulated based on these findings can be instrumental in better preparing for future pandemics.

The concept of resilience has evolved from its origins in engineering resilience to include ecological resilience, evolutionary resilience, and now, pandemic resilience, thereby enriching research on resilience (3). With the study of evolutionary resilience, resilience is no longer only understood as tolerance of external pressures and recovery to the original state; increasing emphasis is being put on stable development of a resilient system, which can achieve self-adaptation and transformation according to its own laws regardless of changes in its external environment (4). At the beginning of the 21st century, as urbanization continues to accelerate, human society is increasingly facing disaster threats that are characterized by high uncertainty and complexity. The traditional disaster governance framework is inadequate to respond to this new context, so resilience theory has been introduced to support disaster responses and management planning for cities and communities (5). Quantifying urban and community resilience by constructing a system of evaluation indicators is an increasingly common approach (6, 7). In response to the need for disaster mitigation under compound hazards, most evaluation indicator systems integrate environmental, infrastructural, economic, social, and institutional resilience into a unified resilience evaluation framework (8–10).

Since the COVID-19 outbreak, the concept of resilience has been increasingly applied to study the pandemic response. Several papers considered the pandemic’s impacts on individuals by examining the psychological resilience of healthcare professionals and the public (11, 12), and some scholars have assessed the organizational resilience of enterprises that survived the pandemic (13, 14). Other scholars have explored anti-pandemic resilience at the national level by examining a country’s performance against COVID-19 from different perspectives such as governance capacity (15, 16), infrastructure development (17–19), economic relief and market volatility (20, 21), community engagement (22, 23), and technology adoption (using technology to improve the response) (24, 25). A few studies have considered COVID-19 as a systematic risk (26) and have integrated the abovementioned dimensions. For example, Haldane et al. (27) constructed a systemic health system resilience framework based on governance and finance, the health workforce, health provision, and community participation to depict the resilience of 28 countries. Chua et al. (28) characterized Singapore’s resilience to the COVID-19 pandemic based on eight core dimensions, including clear leadership and governance, risk communication, health service delivery, and crisis financing. However, these studies were confined to the development of frameworks without implementing quantitative measurements.

Based on our literature review, the existing literature has mainly viewed cities and communities as objects of resilience evaluation, and few resilience studies were conducted at a national or regional level (29). However, COVID-19 has triggered an unprecedented global crisis, and has shifted the resilience perspective from national to regional and global levels. Although some of the literature (15–23) discussed the performance of countries against COVID-19 from a resilience perspective, most focused on only a single dimension, which is inadequate because COVID-19 is a multidimensional test that demands a comprehensive examination of resilience. Although a few studies (26–28) combined multiple resilience dimensions to explore anti-pandemic performance, they still emphasized framework design and were not evaluated in a practical context.

In terms of evaluation methods, resilience assessment has mostly used multi-criteria decision making models, and the empowerment method was used to provide the weights of indicators, with the VIKOR method implemented for the final ranking (30, 31). For example, Abdali et al. (32) constructed an evaluation index system for urban community resilience, then integrated the indices through hierarchical analysis and the VIKOR technique. Sharifi et al. (33) combined Shannon’s entropy with the VIKOR method to rank the resilience of nine neighborhoods in Iran. In summary, the combined empowerment–VIKOR technique is a mainstream method for ranking of evaluation objects, including in the context of disaster resilience evaluation.

Based on this review, we designed the present study to examine anti-pandemic resilience. First, the B&R countries were chosen as research subject, and an evaluation indicator system was constructed to measure their resilience. Next, the weights of the indicators were determined by using the combined empowerment method, and the anti-pandemic resilience of the B&R countries in 2020 was evaluated by using the VIKOR method. We then performed hierarchical clustering to group the countries based on their resilience levels. A consistency test was conducted to compare the actual performance of the countries with the assessed resilience based on our model to verify the validity of the evaluation indicator system. Finally, based on the results of this analysis, policy recommendations were provided to help countries respond better to future pandemics.

The paper is organized as follows: In Section 2, a set of evaluation indicators is constructed and evaluation methods are developed for anti-pandemic resilience assessment. In Section 3, the resilience ranking and clustering results, as well as the validity of the evaluation indicator system, are presented. This validity is confirmed through testing the consistency of actual performance with the assessed resilience. Section 4 analyzes the anti-pandemic resilience of the Belt and Road countries and puts forth policy recommendations to enhance their resilience for future pandemics. Finally, the main conclusions of this study are summarized in Section 5.

## 2. Materials and methods

### 2.1. Data sources and study areas

We focused on the anti-pandemic resilience of the B&R countries in 2020, using data in terms of factors such as the governance capacity, public health preparedness, infrastructure development, national health status, educational attainment, and the state of research and development (R&D) to construct a system of evaluation indicators. The data obtained for each indicator was from the latest datasets available in the Oxford Coronavirus Government Response Tracker (OxCGRT^1^), the COVID-19 Regional Security Assessment database,^2^ the United Nations database,^3^ the World Bank database,^4^ the WHO Global Health Observatory database,^5^ the United Nations Conference on Trade and Development database,^6^ the Human Development Index database,^7^ and the World Intellectual Property Organization statistical database.^8^

**Figure 1:**
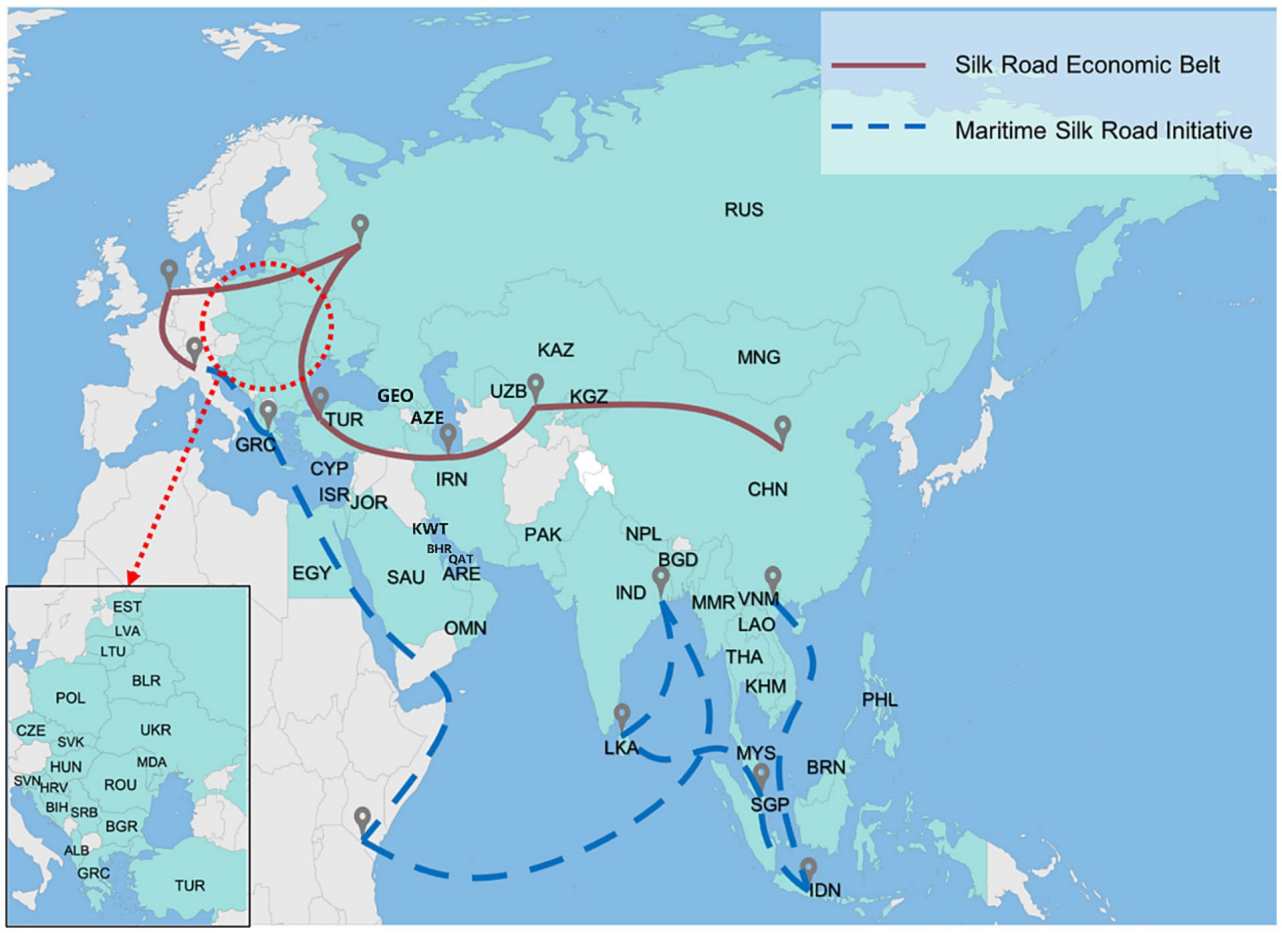
The 53 countries along the Belt and Road route (which comprises the Silk Road Economic Belt and Maritime Silk Road Initiative).

A total of 66 countries lie along the B&R route (34), and we evaluated the anti-pandemic resilience for 53 of the countries for which a complete dataset was available. Of the 13 excluded countries, the real-time outbreak data for Bhutan and Turkmenistan was not included in the World Coronavirus Outbreak Database.^9^ Data on the performance of pandemic prevention policies for Armenia, Montenegro, North Macedonia, Tajikistan, and Maldives was not included in OxCGRT. In addition, six war-torn countries (Yemen, Palestine, Syria, Lebanon, Iraq, and Afghanistan) lacked the most basic medical services, and their volatile situation during the pandemic nearly eliminated their anti-pandemic measures; they were therefore also excluded from this study. Figure 1 shows the study area and the countries along the B&R route. The definitions of all country abbreviations used in Figure 1 are shown in Supplementary Table S1.

### 2.2. Development of an evaluation indicator system for quantifying anti-pandemic resilience

Based on the abovementioned resilience dimensions and the factors that influence resilience, we defined five first-level indicators of resilience: institutional, infrastructural, economic, social, and technological resilience. These first-level indicators were subdivided into 12 s-level indicators and 26 third-level indicators. Table 1 summarizes the evaluation indicator system, and Supplementary Table S2 presents a detailed description of each indicator.

**Table 1:** Evaluation indicator system for the anti-pandemic resilience of countries along the B&R route.

First-level indicator	Second-level indicator	Third-level indicator	Type	Unit of measurement	Source
Institutional resilience	Pandemic management stringency	Government lockdown policy stringency index	+		OxCGRT https://ourworldindata.org/covid-stringency-index
		Public health intervention policy index	+		OxCGRT https://ourworldindata.org/grapher/covid-containment-and-health-index
	Pandemic management efficiency	Government efficiency of risk management index	+		Deep Knowledge Group https://www.dkv.global/covid-safety-assessment-200-regions
Economic resilience	Economic development level	GDP growth rate in the last 5 years	+	%	World Bank https://data.worldbank.org/indicator/NY.GDP.MKTP.KD.ZG
	Poverty level	GDP *per capita*	+	US$	World Bank https://data.worldbank.org/indicator/NY.GDP.PCAP.CD
		The unemployment rate of the adult population	−	%	World Bank https://data.worldbank.org/indicator/SL.UEM.TOTL.NE.ZS
Infrastructure resilience	Healthcare resources and sanitation systems	Medical doctors	+	Per 1,000 people	World Health Organization https://apps.who.int/gho/data/node.main.HWFGRP_0020?lang=en
		Hospital beds	+	Per 1,000 people	World Bank https://data.worldbank.org/indicator/SH.MED.BEDS.ZS
		Nursing and midwifery personnel	+	Per 10,000 people	World Health Organization https://apps.who.int/gho/data/node.main.HWF1?lang=en
		Current health expenditure as percentage of GDP	+	%	World Health Organization https://apps.who.int/gho/data/node.main.GHEDCHEGDPSHA2011?lang=en
		Mortality rate attributable to unsafe water, sanitation and hygiene	−	%	World Health Organization https://apps.who.int/gho/data/node.main.SDG39?lang=en
		Population using at least basic sanitation services	+	%	World Health Organization https://apps.who.int/gho/data/node.main.SDG62?lang=en
	Logistics system	Logistics performance Index	+		World Bank https://lpi.worldbank.org/international/aggregated-ranking?sort=desc&order=Infrastructure#datatable
	Information systems	Population using the internet	+	%	UN database http://data.un.org/Data.aspx?d=ITU&f=ind1Code%3aI99H
		Mobile-cellular telephone subscriptions	+	Per 100 people	UN database http://data.un.org/Data.aspx?d=ITU&f=ind1Code%3aI911
	Energy system	Access to electricity	+	%	World Bank https://data.worldbank.org.cn/indicator/EG.ELC.ACCS.ZS
		Electric power consumption	+	kWh *per capita*	World Bank https://data.worldbank.org/indicator/EG.USE.ELEC.KH.PC
Social resilience	Health level and medical insurance coverage	Life expectancy at birth	+	Years	Human Development Reports http://hdr.undp.org/en/content/latest-human-development-index-ranking
		Universal healthcare service coverage index	+	%	World Health Organization https://apps.who.int/gho/data/node.main.SDG38?lang=en
		Neonatal mortality rate	−	Per 1,000 live births	World Bank https://data.worldbank.org/indicator/SH.DYN.NMRT
	Education level and investment in education	Literacy rate of the adult population	+	%	World Bank https://data.worldbank.org/indicator/SE.ADT.LITR.ZS
		Expected years of schooling	+	Years	Human Development Reports http://hdr.undp.org/en/content/latest-human-development-index-ranking
		Government expenditure on education	+	%	World Bank https://data.worldbank.org/indicator/SE.XPD.TOTL.GD.ZS
Technology resilience	Innovation level and R&D investment	Global Innovation Index	+		WIPO (GII) https://www.wipo.int/global_innovation_index/en/2020/
		Research and development expenditure	+	%	World Bank https://data.worldbank.org/indicator/GB.XPD.RSDV.GD.ZS
	Productive capacity	Productive Capacities Index	+		UNCTAD https://unctadstat.unctad.org/EN/Pci.html

“Type” refers to whether the indicator increases (+) or decreases (−) resilience.

#### 2.2.1. Institutional resilience

The institutional resilience of government is reflected in its ability to organize, manage, and act in the event of a disaster. Numerous studies have demonstrated the effectiveness of non-pharmaceutical interventions undertaken by the government in containing outbreaks (15, 16, 35). In this study, we assess the institutional resilience in terms of pandemic management stringency and efficiency using data from OxCGRT and the COVID-19 Regional Security Assessment Database.

Pandemic management stringency: The lockdown policy stringency index indicates the strictness of government on pandemic prevention and control. The public health intervention policy index represents the government’s policy performance in using health instruments to enable pandemic control. Pandemic management efficiency: The government’s risk management efficiency index reflects the efficiency of government in the face of emergencies.

#### 2.2.2. Infrastructural resilience

COVID-19 has created compounding shocks to national infrastructures (17) in terms of health, logistics, energy, and information systems. The capacity of infrastructure systems to withstand extreme stresses is an important aspect of a country’s resilience to pandemic.

Health system: Adequate health resources and basic sanitation facilities are needed to meet emergency needs during a pandemic (18, 27, 36). The health system indicators we selected are: the numbers of doctors, beds, and nurses (including midwives) *per capita*, which reflect the medical relief capacity during a pandemic. The share of GDP accounted for by health care expenditures, which reflects the priority given to health care by the national government. The mortality rate due to unsafe water, sanitation and hygiene, and the number of people covered by basic sanitation services reflect the adequacy of the national hygiene facilities.

Logistics system: Logistics represents the role of marshaling and distributing goods, and is critical for the continued functioning of the supply chain (37). The logistics performance index measures the national logistics development and reflects the ability to deploy human and material resources during the pandemic.

Energy system: The energy system is critical to maintaining the order of daily life and the functioning of critical sectors (especially for hospitals and factories) (19, 38). The coverage of a country by the electrical grid and electric power consumption can reflect the resilience of a country’s energy system.

Information systems: Information technology facilitates the distribution of information and plays important roles in tracking and detection, telemedicine, and home office work (25, 39). We measure the ability to use the information technology to mitigate the impact of the pandemic through two indicators: the proportion of the population using the Internet and the percentage of the population that uses a cell phone.

#### 2.2.3. Economic resilience

The level of economic development reflects the national economic basis to withstand the shock, whilst poverty may cause a country to be more vulnerable to pandemic shocks (20, 21). In this paper, we chose two second-level indicators: the level of economic development and the level of poverty. For the level of economic development, we used the economic growth rate in the past 5 years to reflect the growth rate of the country’s total economy and overall economic strength For the level of poverty, we used the GDP *per capita* and unemployment rate to measure the average living standard of the population and the scale of economically vulnerable groups.

#### 2.2.4. Social resilience

In the face of an epidemic, it is crucial for a citizens to be in a good shape and equip with sufficient knowledge to know what is happening and how they can respond (22, 40). We chose two second-level indicators under social resilience: the health level and medical insurance coverage, and the education level and investment in education.

Health level and medical insurance coverage: Life expectancy *per capita* and the neonatal mortality rate reflect the overall health status of citizens. Universal healthcare reflects the state’s coverage of basic health services required by citizens (41). Education level and investment in education: The expected years of schooling and literacy rate reflect the development of education. The education expenditure reflects the importance the national government attaches to education.

#### 2.2.5. Technological resilience

Technological resilience reflects a country’s ability to adapt to disasters as quickly as possible. The most powerful weapons in the human battle against disease are science and technology, since the ability to develop (science) and produce (technology) vaccines to overcome viruses is essential for the early response and subsequent recovery (42). We chose two second-level indicators, namely the level of R&D and the production capacity, to measure the technological resilience.

For the level of R&D, we used the R&D expenditure and the global innovation index to reflect the country’s scientific and technological strengths and innovation performance. For the production capacity, we used the productive capacity index from the UNCTAD database to reflect a country’s ability to produce goods and services.

### 2.3. Evaluation methods for anti-pandemic resilience assessment

The previous section described the inputs for developing the evaluation indicator system. In this section, we determine the weight of each indicator. To do so, we integrate a weighting method with a three-scale method and the improved entropy method, making full use of objective information but also accounts for subjective choices by decision-makers.

#### 2.3.1. Determination of weights

(1) Determining the weight of the first-level indicators using the three-scale method

The three-scale method is used to perform pairwise comparisons between indicators. Here, using the 1–3 scales method instead of 1–9 scales can make it easier to accurately determine the judgment matrix and to meet the consistency requirements for integrating multiple indicators (43). We used the following method to integrate the weights of the five first-level indicators that we chose:

We used the three-scale method (with values of 0, 1, or 2) to perform pairwise comparisons of the five indicators, and established a comparison matrix to calculate the rank of each indicator. We used **D** = (*d_ij_*) = {0,1,2} to represent the judgment scale set, where *d_ij_* = 0 means that eigenfactor *i* is less important than eigenfactor *j*, *d_ij_* = 1 means that eigenfactor *i* is as important as eigenfactor *j*, and *d_ij_* = 2 means that eigenfactor *i* is more important than eigenfactor *j*.

With 2020 being the first year of the fight against COVID-19 in most countries, breaking the chain of virus transmission was particularly critical in the absence of an effective vaccine. Non-pharmaceutical interventions such as lockdowns, quarantine, wearing masks, and tracking have proven to be the most effective means of pandemic control (44, 45), so we considered institutional resilience (IR) to be the most important indicator. Technological resilience (TR) refers to the level of R&D and productive capacity, which is a proxy for the development and production of vaccines. However, since we focused on national anti-pandemic performance in 2020, when vaccines were not yet widely available (46), we considered TR to be the least important indicator. We propose that infrastructure resilience (FR), economic resilience (ER), and social resilience (SR) were also important dimensions for mitigating the pandemic, with intermediate values between IR and TR, and were equally important. Table 2 shows the resulting pairwise comparisons.

**Table 2:** Comparison matrix for the eigenfactors of the five first-level indicators.

	IR	FR	SR	ER	TR	*b_i_*
IR	1	2	2	2	2	9
FR	0	1	1	1	2	5
ER	0	1	1	1	2	5
SR	0	1	1	1	2	5
TR	0	0	0	0	1	1

IR, institutional resilience; FR, infrastructural resilience; ER, economic resilience; SR, social resilience; TR, technological resilience; and b_i_ equals the sum of the values in each row.

We constructed the judgment matrix using the range method. In this method, *y_ij_* denotes the ratio of the importance of eigenfactor *i* to eigenfactor *j*. The ratio of the importance of eigenvector *j* to that of eigenfactor *i* is then 1/*y_ij_*. According to the range method, *f*(*b_i_*, *b_j_*) = *y_ij_* = $y_{b}^{\frac{b_{i}-b_{j}}{B}}$, and **Y** = (*y_ij_*) is the consistency judgment matrix. *y_b_* is the relative importance of the range element pairs, and is predetermined, and is generally assigned a constant value of *y_b_* = 9 based on a certain standard (47). *B* = max (*b*_1_, *b*_2_, *b*_3_, *b*_4_, *b*_5_) – min (*b*_1_, *b*_2_, *b*_3_, *b*_4_, *b*_5_), and equals the range, so *B* = 8 in this paper based on the values in Table 2. Accordingly, the eigenfactor judgment matrix is then obtained as follows:


(1) $$\begin{array}{l} Y=\left(y_{ij}\right)=\\ \left[\begin{array}{ccccccccc} y & \mathrm{IR} & \mathrm{FR} & ER & SR & TR & M_{i} & W_{i} & \bar{W_{i}}\\ \mathrm{IR} & 1 & 3 & 3 & 3 & 9 & 243 & 3 & 0.474\\ \mathrm{FR} & 0.333 & 1 & 1 & 1 & 3 & 10 & 1 & 0.158\\ ER & 0.333 & 1 & 1 & 1 & 3 & 10 & 1 & 0.158\\ SR & 0.333 & 1 & 1 & 1 & 3 & 10 & 1 & 0.158\\ TR & 0.111 & 0.333 & 0.333 & 0.333 & 1 & 0.041 & 0.333 & 0.053 \end{array}\right] \end{array}$$

Where


(2) $$M_{i}=\overset{5}{\underset{j=1}{\prod }}y_{ij}$$

(3) $$W_{i}=\sqrt[{5}]{M_{i}},\overset{5}{\underset{i=1}{\sum }}W_{i}=6.333$$

(4) $$\bar{W_{i}}=\frac{W_{i}}{\sum_{k=1}^{5}W_{k}}\left(i=1,2,\ldots ,5\right)$$

(5) $$\overset{5}{\underset{i=1}{\sum }}\bar{W_{i}}=1$$

The weights of the five first-level indicators are included in the transform matrix ${\bar{W}}^{T}=\left(0.474,0.158,0.158,0.158,0.053\right)$ for IR, FR, ER, SR, and TR, respectively.

If **C** is a partial matrix of **Y** that contains the first five columns of **Y**, then **L** = (*l_i_*)_5 × 1_ = **C·W**^T^ = (2.164, 0.721, 0.721, 0.721, 0.240)^T^. The maximum eigenvalue is $\lambda_{\max}=\frac{1}{5}\times \overset{5}{\underset{i=1}{\sum }}\frac{l_{i}}{W_{i}}=5$, and $P_{\mathrm{CI}}=\frac{{}_{\lambda \max}-5}{5-1}=0\leq \;\;\varepsilon \left(\varepsilon =0.001\right)$ satisfies the consistency test. Therefore, the weights of the five first-level indicators obtained by the three-scale method are $\left(\bar{W_{1}},\bar{W_{2}},\bar{W_{3}},\bar{W_{4}},\bar{W_{5}}\right)=\left(0.474,0.158,0.158,0.158,0.053\right)$ for IR, FR, ER, SR, and TR, respectively.

(2) Determining the weight of the second-level indicators using the arithmetic mean method

Next, we assigned weights to the second-level indicators based on the weights of the first-level indicators. Let the set **C***_i_* denote all the second-level indicators corresponding to the *i-*th first-level indicator, and let *w_ij_* denote the weight of the *j-*th second-level indicator. The formula for calculating the weights of the second-level indicators is then:

(6) $$w_{ij}=\frac{\bar{W_{i}}}{\left|C_{i}\right|}$$

Where $\bar{W_{i}}$ denotes the weight of the *i-*th first-level indicator, and |**C***_i_*| denotes the number of all of all second-level indicators in set **C***_i_*. It is difficult for us to objectively judge the differences in the importance of secondary indicators, so we assigned the weights of the second-level indicators (*w_ij_*) by equally dividing $\bar{W_{i}}$ among the second-level indicators.

(3) Determining the weight of the third-level indicators using the improved entropy-weight method

We used the improved entropy-weight method to measure the weights of the third-level indicators, using the following method:

Establish an anti-pandemic resilience assessment matrix for countries along the B&R route. We collected data for the indicator system and constructed the original matrix **A**, which contains data from the 53 B&R countries for the 26 resilience evaluation indicators. The set of resilience evaluation indicators is denoted as {*A*_1,_ *A*_2, …,_ *A*_26_}, and *a_q_* denotes the *q-*th indicator for *q* = 1 to 26 and *p* represents countries 1 to 53:


(7) $$A=\left(\begin{array}{ccccc} a_{11} & \cdots & a_{1q} & \cdots & a_{1,26}\\ \vdots & \vdots & \vdots & \vdots & \vdots \\ a_{p1} & \cdots & a_{pq} & \cdots & a_{p,26}\\ \vdots & \vdots & \vdots & \vdots & \vdots \\ a_{53,1} & \cdots & a_{53,q} & \cdots & a_{53,26} \end{array}\right)={\left(a_{pq}\right)}_{53\times 26}$$

Where *a_pq_* represents the value of the *q-*th indicator for the *p*-th country, and the indicators are transformed differently according to the characteristics of the indicator *a_pq_*:

For benefit indicators:


(8) $$x_{pq}=\left\{\begin{array}{c} \frac{a_{pq}-m_{q}^{-}}{m_{q}^{+}-m_{q}^{-}},\;\;m_{q}^{+}\neq m_{q}^{-}\\ 1,\;\;m_{q}^{+}=m_{q}^{-} \end{array}\right.$$

For cost indicators:


(9) $$x_{pq}=\left\{\begin{array}{c} \frac{m_{q}^{+}-a_{pq}}{m_{q}^{+}-m_{q}^{-}},\;m_{q}^{+}\neq m_{q}^{-}\\ 1,\;m_{q}^{+}=m_{q}^{-} \end{array}\right.$$

Where $m_{q}^{+}$ denotes the maximum value in the *q-*th column of the matrix:$\;m_{q}^{+}=\max\left(a_{pq}\right),p=1,2,\ldots ,53.m_{q}^{-}$ denotes the minimum value in the *q-*th column: $m_{q}^{-}=\min\left(a_{pq}\right),p=1,2,\ldots ,53$.

Thus, the decision matrix **X** is created:


(10) $$X=\left(\begin{array}{ccccc} x_{11} & \cdots & x_{1q} & \cdots & x_{1,26}\\ \vdots & \vdots & \vdots & \vdots & \vdots \\ x_{p1} & \cdots & x_{pq} & \cdots & x_{p,26}\\ \vdots & \vdots & \vdots & \vdots & \vdots \\ x_{53,1} & \cdots & x_{53,q} & \cdots & x_{53,26} \end{array}\right)={\left(x_{pq}\right)}_{53\times 26}$$

Transform the decision matrix into dimensionless data, resulting in the standardized decision matrix **X’**.


(11) $$X^{\prime }=\left(\begin{array}{ccccc} x_{11}^{\prime } & \cdots & x_{1q}^{\prime } & \cdots & x_{1,26}^{\prime }\\ \vdots & \vdots & \vdots & \vdots & \vdots \\ x_{p1}^{\prime } & \cdots & x_{pq}^{\prime } & \cdots & x_{p,26}^{\prime }\\ \vdots & \vdots & \vdots & \vdots & \vdots \\ x_{53,1}^{\prime } & \cdots & x_{53,q}^{\prime } & \cdots & x_{53,26}^{\prime } \end{array}\right)={\left(x_{pq}^{\prime }\right)}_{53\times 26}$$

Where $$\;x_{pq}^{\prime }=\frac{x_{pq}}{\sum_{i=1}^{53}x_{iq}},p=1,2,\ldots ,53;q=1,2,\ldots ,26$$

Calculate the entropy value (*E_q_*) and the weights of the third-level indicators:


(12) $$E_{q}=-\frac{1}{\ln53}\overset{53}{\underset{p=1}{\sum }}\left(x_{pq}^{\prime }\times \ln x_{pq}^{\prime }\right)\;\;\left(q=1,\ldots ,26\right)$$

The set of indicators corresponding to the data matrix is {**A**_1_, **A**_2_, …, **A***_q_*, … **A**_26_}, and **C***_ij_* is the set of third-level indicators corresponding to the *j-*th second-level indicator. If the indicators in column **A***_q_* of the data matrix (i.e., the third-level indicators) belong to the set, then:


(13) $$\begin{array}{c} w_{q,ij}=\frac{1-E_{q}}{\sum_{A_{q}\in C_{ij}}\left(1-E_{q}\right)}\;\;\left(q=1,\ldots ,26\right) \end{array}$$

(14) $$\begin{array}{c} \underset{A_{q}\in C_{ij}}{\sum }w_{q,ij}=1\;\;\left(q=1,\ldots ,26\right) \end{array}$$

Where *w_q,ij_* denotes the weights of the third-level indicators corresponding to the *j-*th second-level indicator, and the indicators in column **A***_q_* (the third-level indicators) belong to the set **C***_ij_*.$\;\sum_{A_{q}\in C_{ij}}w_{q,ij}$ represents the sum of the weights of all third-level indicators under the *j-*th second-level indicator.

Calculate the final weights of the third-level indicators in the column **A***_q_*:

The final weight *w_q_* of each third-level indicator in the column **A***_q_* is the entropy weight of the third-level indicator calculated in the previous step multiplied by the weight of its corresponding second-level indicator.


(15) $$\begin{array}{c} w_{q}=w_{q,ij}\;\cdot \;w_{ij}\;\;\left(s.t.A_{q}\in C_{ij}\right) \end{array}$$

Where *w_ij_* represents the weight of the *j*-th second-level indicator under the *i*-th first-level indicator, which is obtained as described in Section 3.1.2. *w_q,ij_* represents the weight of the third-level indicator under the *j-*th second-level indicator, and **A***_q_* ∈ **C***_ij_*, which is obtained by the improved entropy-weight method (Section 3.1.3). In summary, the final weights *w_q_* of the 26 third-level indicators can be obtained using Equation (15).

#### 2.3.2. Evaluate a country’s anti-pandemic resilience using the VIKOR method

The TOPSIS and VIKOR methods have both been introduced as applicable techniques for implementation in multi-criteria decision-making. However, TOPSIS cannot account for the utility of a given solution to the group or for the individual regrets. VIKOR overcomes that shortcoming to arrive at a more rational decision (48). Therefore, to assess the anti-pandemic resilience for countries along the B&R route, we used the VIKOR method to rank each country’s resilience by building an evaluation indicator system and setting indicator weights. The calculation process is as follows.

First, we standardize the original matrix **A** so that the values of all indicators are within [0, 1]:


(16) $$a_{pq}^{\prime }=\frac{a_{pq}-\underset{q}{\min}a_{pq}}{\underset{q}{\max}a_{pq}-\underset{q}{\min}a_{pq}}$$

Where *a_pq_* is the value for the *p*-th country and *q*-th indicator (*p* = 1 to 53 and *q* = 1 to 26), and max and min represent the maximum and minimum values for the *q*-th indicator.

Next, we use the dimensionless $a_{pq}^{\prime }$ to obtain *f_pq_* as follows:


(17) $$\begin{array}{c} f_{pq}=\frac{a_{pq}^{\prime }}{\sqrt{\sum_{k=1}^{53}{\left(a_{kq}^{\prime }\right)}^{2}}}\left(q=1,\ldots ,26\right) \end{array}$$

We then determine the positive and negative ideal solutions for each attribute as $f_{q}^{+}$ and $f_{q}^{-}$:


(18) $$f_{q}^{+}=\left[\left(\underset{p}{\max}f_{pq}|q\in J_{1}\right),\left(\underset{p}{\min}f_{pq}|q\in J_{2}\right)\right]$$

(19) $$f_{q}^{-}=\left[\left(\underset{p}{\min}f_{pq}|q\in J_{1}\right),\left(\underset{p}{\max}f_{pq}|q\in J_{2}\right)\right]$$

Where **J**_1_ is the set of benefit-based indicators and **J**_2_ is the set of cost-based indicators.

Next, we calculate the distance to the ideal solution for each scheme:

First, we calculate the population utility *S_p_* and the individual regret *R_p_* for each scheme, where *S_p_* represents the weighted distance of the *p-*th scheme from the ideal solution and *R_p_* represents the maximum distance of the *p-*th scheme from the positive ideal solution:


(20) $$S_{p}=\overset{26}{\underset{q=1}{\sum }}w_{q}\frac{\left(f_{q}^{+}-f_{pq}\right)}{f_{q}^{+}-f_{q}^{-}},p=1,\ldots ,53$$

(21) $$R_{p}=\underset{q}{\max}\left[w_{q}\left(f_{q}^{+}-f_{pq}\right)/\left(f_{q}^{+}-f_{q}^{-}\right)\right],p=1,\ldots ,53$$

where *w_q_* denotes the final weight of the third-level indicator **A***_q_* that we obtained in the previous section.

Afterwards, we calculate the holistic value *Q_p_* for each country based on group (population) utility and individual regret:


(22) $$Q_{p}=V\left[\frac{S_{p}-S^{+}}{S^{-}-S^{+}}\right]+\left(1-V\right)\left[\frac{R_{p}-R^{+}}{R^{-}-R^{+}}\right]$$

where both the group utility *S_p_* and the individual regret *R_p_* are cost attributes, and their positive and negative ideal solutions are *S*^+^ = min(*S_p_*), *S*^−^ = max(*S_p_*), *R*^+^ = min(*R_p_*), and *R*^−^ = max(*R_p_*) for *p* = 1 to 53. *V* represents the degree of preference for maximizing group utility and minimizing individual regret. A value of *V* greater than 0.5 indicates a preference for maximizing group utility, whereas a value less than 0.5 indicates a preference for minimizing individual regret. Here, we set *V* = 0.5 to indicate no preference.

Finally, we defined the anti-pandemic resilience (*U_p_*) using Equation 23. The value of resilience ranges from 0 to 1, and the higher the value, the higher the resilience.


(23) $$U_{p}=1-Q_{p}$$

#### 2.3.3. Classify levels of anti-pandemic resilience using the hierarchical clustering method

We performed hierarchical clustering of the 53 B&R countries based on the evaluated resilience value up obtained by the VIKOR method described in the previous section. We classified the countries into different levels according to the clustering results to analyze the resilience performance with different levels.

In the first step, we applied hierarchical clustering to cluster the resilience performance of the 53 countries along the B&R route. We used version 22.0 of the SPSS software^10^ to calculate the distance between data points based on the sum of squares of the deviations (Ward’s method), using the *U_p_* value obtained above as the analysis variable. In this approach, data points that are close to each other are grouped into one category as much as possible, so that the distance within a given category is smaller and the distance between different categories is larger. The calculation is performed iteratively, adding one data point at a time, until all data points are completely grouped into one category. The corresponding clustering spectrum can then be graphed.

In the second step, we calculated the silhouette coefficients to determine the optimal clustering number for the 53 countries (49). The silhouette coefficient *S*(*i*) combines two factors: cohesion *a*(*i*) and dispersion *b*(*i*). *a*(*i*) represents the average distance between object *i* and the other objects in the same cluster, and *b*(*i*) represents the average distance between object *i* and all objects in other clusters. The silhouette coefficient takes values in [−1, 1], and the closer the value is to 1, the more compact the objects in the cluster are to each other, the larger the distance between clusters, and the better the clustering effect. We therefore chose the number of categories when the silhouette coefficient was closest to 1 as the final clustering number:


(24) $$S\left(i\right)=\frac{\left[b\left(i\right)-a\left(i\right)\right]}{\max\left\{a\left(i\right),b\left(i\right)\right\}}$$

## 3. Results

### 3.1. Resilience ranking and clustering for countries along the B&R route

Based on the VIKOR results, we ranked the resilience of the B&R countries in 2020 using *U_p_* (Table 3). The five most resilient countries were Israel, Bahrain, China, Singapore, and the UAE; the five least resilient were Kyrgyzstan, Iran, Myanmar, the Lao PDR, and Cambodia.

**Table 3:** Ranking of the Belt and Road countries based on anti-pandemic resilience (*U_p_*); low ranks and *U_p_* values represent high resilience.

Rank	Country	*U_p_*	Rank	Country	*U_p_*
1	Israel	1	28	Ukraine	0.2868
2	Bahrain	0.9107	29	Romania	0.2753
3	China	0.9078	30	Bulgaria	0.2561
4	Singapore	0.9026	31	Albania	0.2524
5	United Arab Emirates	0.8859	32	Thailand	0.2517
6	Oman	0.8599	33	Azerbaijan	0.2497
7	Qatar	0.8570	34	Serbia	0.226
8	Saudi Arabia	0.8558	35	Jordan	0.2257
9	Kuwait	0.8465	36	Philippines	0.2135
10	Cyprus	0.7786	37	Kazakhstan	0.2068
11	Hungary	0.67	38	Mongolia	0.2033
12	Estonia	0.6443	39	Belarus	0.1875
13	Vietnam	0.6363	40	Indonesia	0.1859
14	Greece	0.6033	41	Sri Lanka	0.1363
15	Poland	0.6027	42	Moldova	0.1315
16	Slovenia	0.5922	43	Pakistan	0.1289
17	Turkey	0.5697	44	Bangladesh	0.1224
18	Lithuania	0.5601	45	Nepal	0.1208
19	Latvia	0.5286	46	Uzbekistan	0.117
20	Russia	0.5122	47	Egypt	0.1159
21	Georgia	0.4837	48	Bosnia and Herz.	0.1119
22	Czech Rep.	0.4624	49	Kyrgyzstan	0.1103
23	Brunei	0.4427	50	Iran	0.0852
24	Malaysia	0.4101	51	Myanmar	0.0543
25	Croatia	0.3398	52	Lao PDR	0.0206
26	Slovakia	0.3372	53	Cambodia	0
27	India	0.3367			

**Figure 2:**
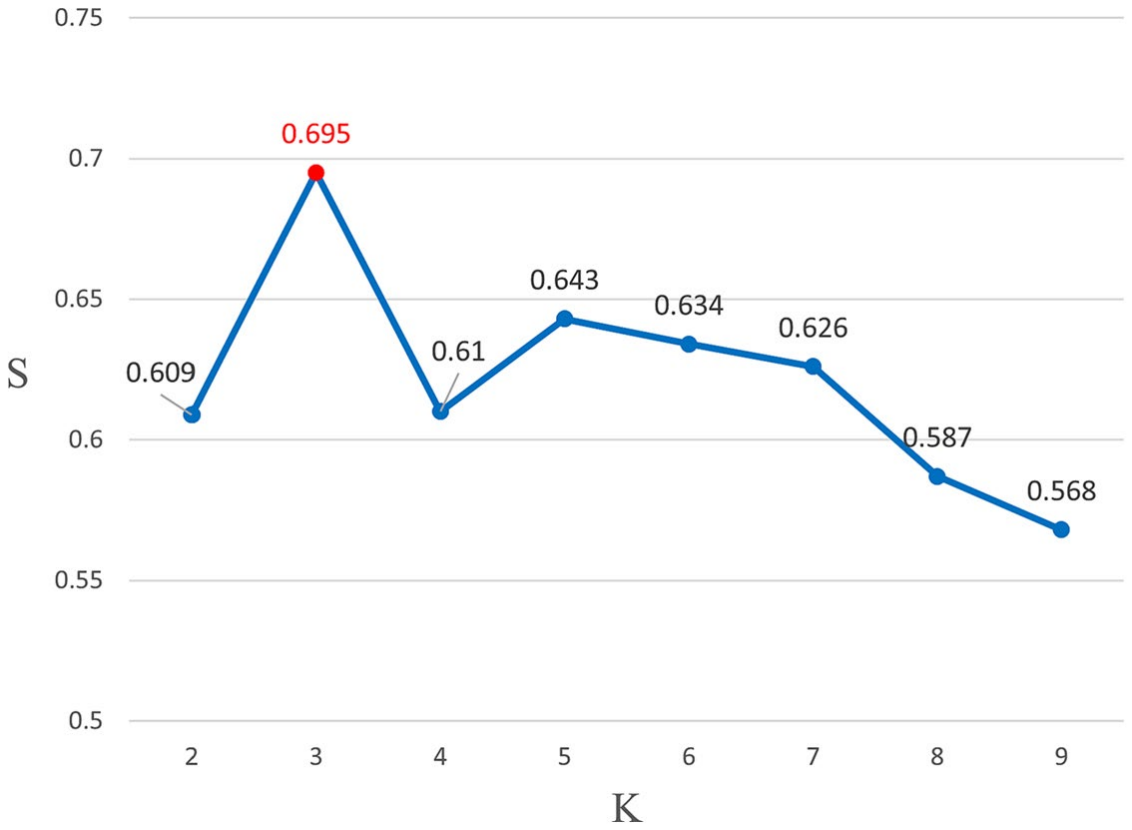
Silhouette coefficient (*S*) diagram for *K* clusters.

The effectiveness of the different clusters was assessed by analyzing the clustering silhouette coefficients S(i). Figure 2 shows that the silhouette coefficient reached its maximum value of 0.659 with *K* = 3 clusters, so this represents the optimal clustering. Therefore, we divided the 53 B&R countries into three categories and analyzed the anti-pandemic resilience in these categories.

**Figure 3:**
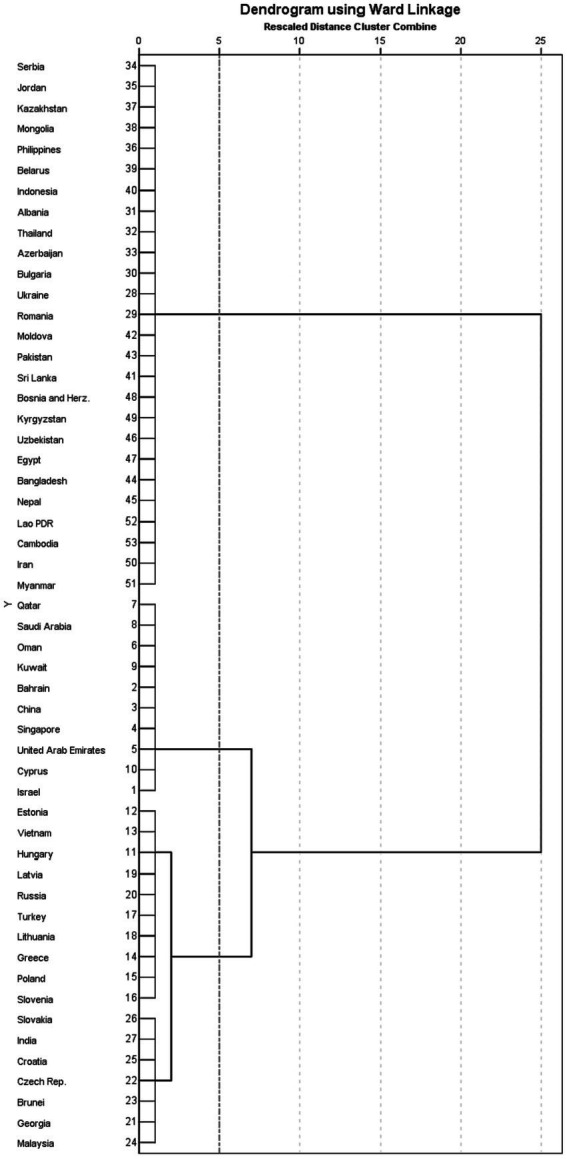
Cluster analysis spectrum chart. Numbers on the *y*-axis represent the resilience ranking shown in Table 3.

Figure 3 shows the clustering diagram with three clusters. The 53 countries along the B&R route can be classified into groups with high resilience (*U_p_* > 0.7), moderate resilience (0.3 < *U_p_* < 0.7), and low resilience (*U_p_* < 0.3) levels based on *U_p_* (Table 3). Table 4 summarizes the countries within each group in order of resilience.

**Table 4:** Anti-pandemic resilience levels of the 53 countries along the B&R route.

Resilience level	Countries
High resilience (*U_p_* > 0.7)	Israel, Bahrain, China, Singapore, United Arab Emirates, Oman, Qatar, Saudi Arabia, Kuwait, Cyprus
Moderate resilience (0.3 < *U_p_* < 0.7)	Hungary, Estonia, Vietnam, Greece, Poland, Slovenia, Turkey, Lithuania, Latvia, Russia, Georgia, Czech Rep., Brunei, Malaysia, Croatia, Slovakia, India
Low resilience (*U_p_* < 0.3)	Ukraine, Romania, Bulgaria, Albania, Thailand, Azerbaijan, Serbia, Jordan, Philippines, Kazakhstan, Mongolia, Belarus, Indonesia, Sri Lanka, Moldova, Pakistan, Bangladesh, Nepal, Uzbekistan, Egypt, Bosnia and Herz., Kyrgyzstan, Iran, Myanmar, Lao PDR, Cambodia

Countries are listed from most to least resilient within a given category.

**Figure 4:**
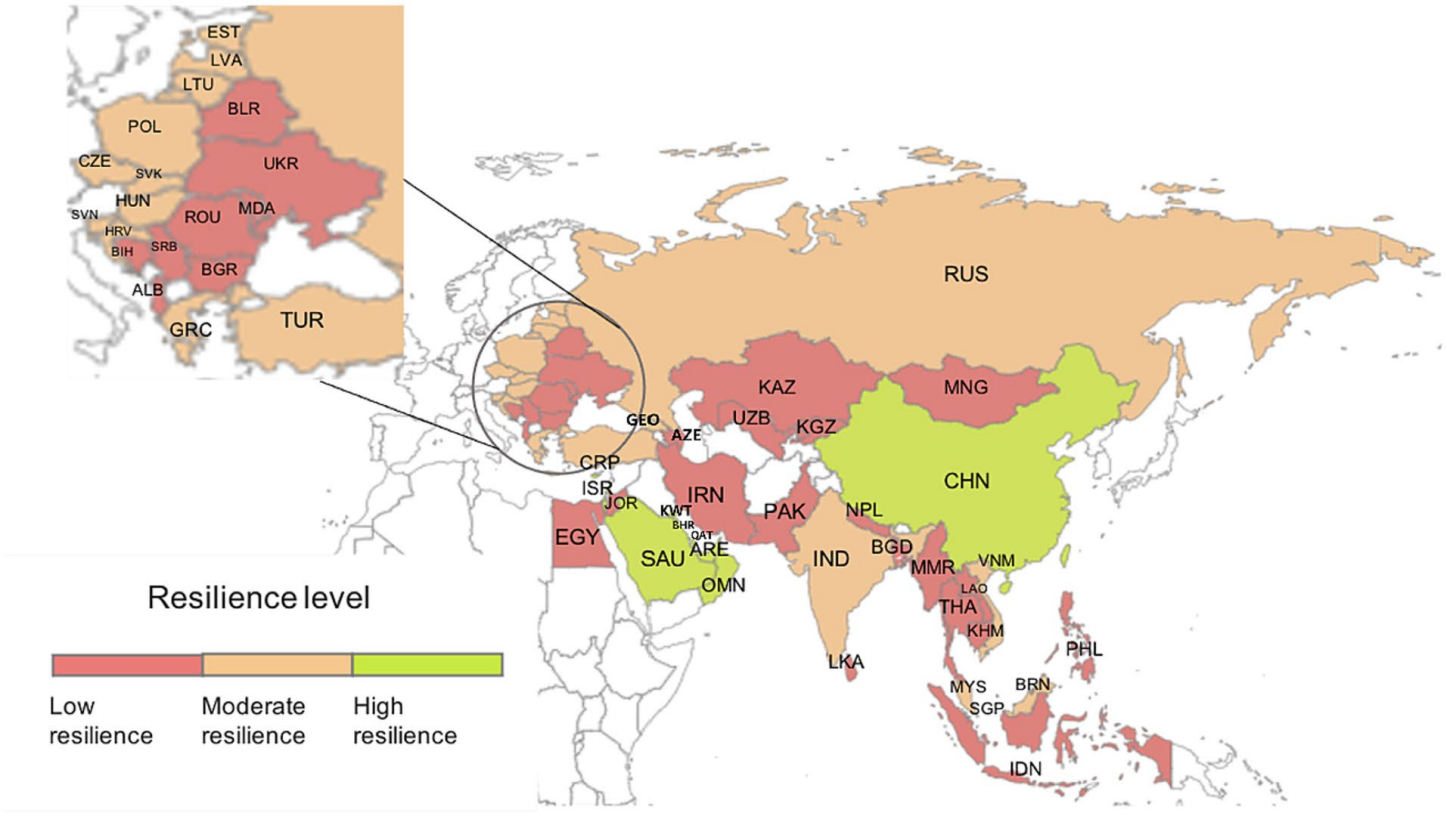
Spatial distribution of anti-pandemic resilience of the 53 Belt and Road countries. Resilience levels are based on the data in Table 4.

We clarified the spatial pattern of the resilience of the countries along the B&R route in a map of the region (Figure 4). The former Soviet Bloc countries in eastern Europe were most likely to have low resilience, but many countries in southeast Asia also had low resilience.

### 3.2. Consistency between the assessed and actual anti-pandemic resilience

In the previous section, we derived the anti-pandemic resilience of the B&R countries using the evaluation indicator system developed in this study. To test the validity of the system, we collected actual data from the B&R countries in 2020 to test the consistency between the actual and the assessed resilience.

We combined three indicators to reflect a country’s actual resilience: the cumulative number of confirmed COVID-19 diagnoses per million population, the number of deaths per million population, and the monitoring and detection scores for the year 2020 (50). We obtained this data from the World Coronavirus Outbreak Real-Time Data website (see text footnote 9) and the COVID-19 Regional Security Assessment database^11^ (Supplementary Table S3). We assigned equal weights to the three indicators and calculated the actual anti-pandemic resilience assessment value $U_{p}^{\prime }$ of the B&R countries using the VIKOR method. Supplementary Table S4 presents the actual resilience performance of each country.

**Figure 5:**
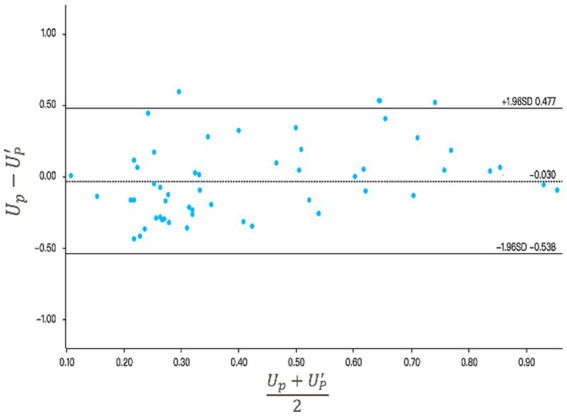
Analysis of the consistency between the assessed anti-pandemic resilience (*U_p_*) and the actual anti-epidemic resilience ($U_{p}^{\prime }$) using the Bland–Altman method. The center line represents the mean difference; the upper and lower lines represent the 95% confidence interval.

We used a Bland–Altman plot to verify the consistency between the assessed resilience value *U_p_* and the actual resilience value $U_{p}^{\prime }$. This plot quantifies the consistency between two datasets by examining the mean difference and constructing boundaries for the 95% confidence interval. If most data points fall within the confidence interval, then this indicates a good level of consistency between the two datasets (51). Figure 5 shows the results of this analysis. The mean value of the difference between the assessed and actual resilience was −0.03, and the 95% confidence interval for this difference was (−0.538, 0.477). Most data points fell within the 95% confidence interval, with only three outliers. Thus, our assessment method provides values that show good consistency with the actual results.

## 4. Discussion

### 4.1. Analysis of the anti-pandemic resilience in the B&R countries

**Figure 6:**
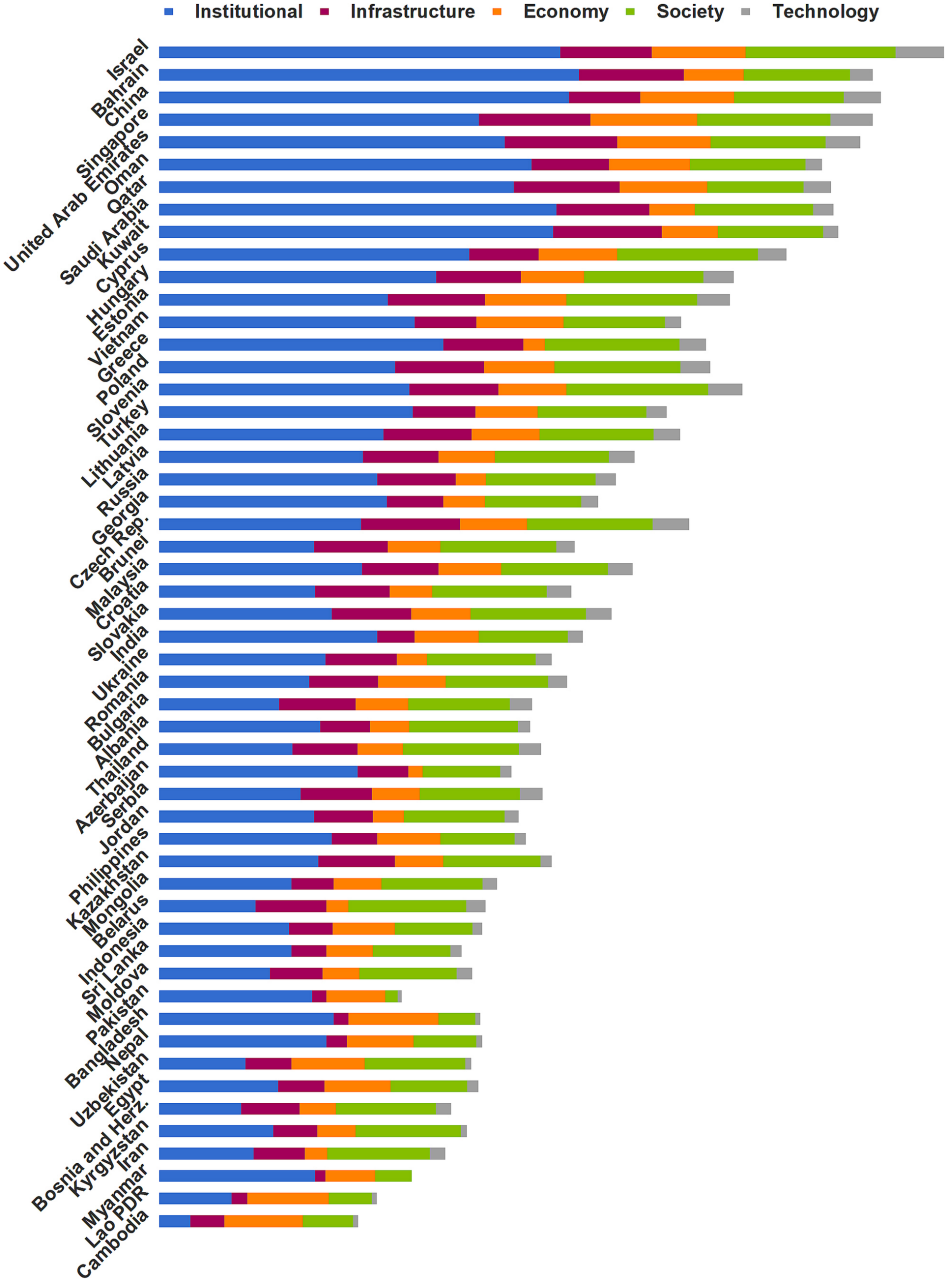
Resilience performance for the 53 Belt and Road countries based on the five first-level dimensions.

In this section, a comparative analysis of national resilience is conducted based on the five first-level indicators, namely institutional, infrastructure, economic, social, and technological resilience. We quantified the resilience by calculating the scores of each country for each indicator after standardizing the indicator values using Equations 8, 9, then multiplied the standardized values by the indicator weights (*w_q_*) obtained from Equation 15 to obtain the resilience score for each indicator. Figure 6 shows the results, and Supplementary Table S5 provides the numerical contributions of each of the five indicators for each country.

**Figure 7:**
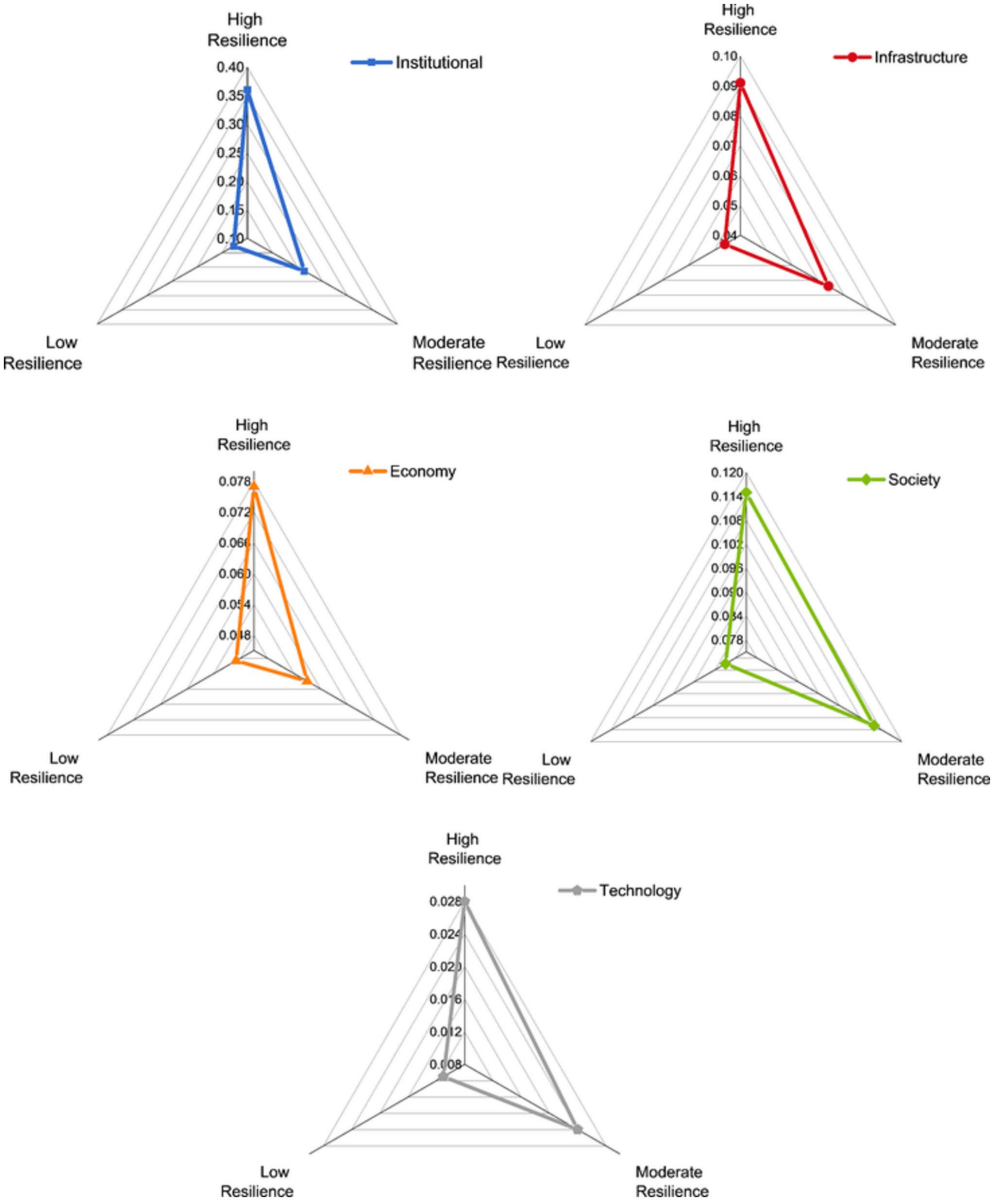
Scores of the five first-level indicators for the country groups with high, moderate, and low anti-pandemic resilience.

We classified the B&R countries as having high, moderate, or low resilience. Figure 7 shows the scores of these three levels for each of the five first-level resilience indicators. The countries with high resilience benefited from a combination of institutional, infrastructural, economic, social, and technological resilience to contain the pandemic. Most with moderate resilience did not differ greatly from those with high resilience in terms of social, technological, and infrastructural resilience, but had lower institutional and economic resilience. Countries with low resilience performed poorly in all five dimensions of resilience due to their weak health systems, poor economy, and inefficient response.

We gained several insights from considering the factors that fostered strong resilience in 2020. Countries with strong resilience had high institutional resilience scores (an average of 0.360), and those with moderate resilience were less rigorous or consistent in their efforts to control the pandemic, with an average institutional resilience score of 0.214. The low-resilience countries responded ineffectively, with an average institutional resilience score of 0.127. Bahrain (0.406) ranked first in pandemic prevention intensity by continuously upgrading the pandemic level and extending quarantines and lockdowns, whereas China (0.396) ranked first in pandemic prevention efficiency by limiting spread of the virus most promptly. Among countries with moderate resilience, Greece (0.275) responded early in the pandemic, mitigating the subsequent impact on the health system to some extent. Vietnam (0.247) had among the highest preparednesses for its accumulated experience with previous infectious respiratory diseases. Russia (0.211), on the other hand, responded to the pandemic with weak and incoherent prevention measures (52). Innovative public health surveillance systems provide stakeholders with actionable information to limit disease risk earlier (53). We found that the detection capacity in most of the low-resilience countries could not keep pace with spread of the virus, and the limited surveillance prevented the government from finding the source of an infection timely. This led to low scores for countries such as Cambodia (0.030) and Laos (0.070). The government of Belarus (0.093) even officially denied the existence of a pandemic initially and did not intervene to prevent mass gatherings that spread the infection (52). In contrast, the Philippines (0.167), Kazakhstan (0.154), and Pakistan (0.148) confronted the weakness of their health systems and took rigorous measures to avoid mass outbreaks before the virus arrived (54).

In terms of infrastructure resilience, the mean resilience scores for countries with high and moderate resilience were 0.091 and 0.074, respectively. In contrast, countries with low resilience lagged far behind, with a mean score of 0.046. The UAE (0.109) and Singapore (0.108) outperformed. Singapore implemented a medical stockpile that could last 6 months after learning from the 2002 SARS outbreak (55), while strong logistic, information, and energy systems in the UAE provided a solid foundation to control the virus’ spread. In contrast, Oman (0.075), China (0.069), and Cyprus (0.067) lagged behind for their relatively limited medical and energy resources available *per capita*. As a representative of countries with moderate resilience, the Czech Republic (0.096) and Estonia (0.094) invested strongly in their healthcare systems. Additionally, the Czech Republic had a well-developed logistics system that could support the distribution of emergency supplies, and Estonia’s information systems were widespread, thereby meeting the needs of working from home and remote socialization. In contrast, India’s infrastructure resilience score (0.036) was far below average. Relative to large population, its healthcare workers and beds were in short. What is worse, a severe lack of sanitation facilities exacerbated the situation and facilitated the spread of viruses. In addition, Georgia (0.055) performed poorly due to its underdeveloped logistics system and low energy resources. The low-resilience countries are unable to respond to the high volume and diversity of demand in normal times and were even less able to cope with a pandemic. For example, the public healthcare system in Myanmar (0.010) was completely unable to withstand the pandemic (56). In Pakistan (0.014) and Bangladesh (0.014), health care has not achieved universal coverage, and many poor families could not seek medical care because of the high cost, exacerbating spread of the virus (57).

In terms of economic resilience, countries with high, moderate, and low resilience obtained mean scores of 0.077, 0.057, and 0.049, respectively. Among countries with high resilience, Cyprus (0.076), Bahrain (0.058), Kuwait (0.054), and Saudi Arabia (0.044) scored below average. These countries heavily relied on energy exports, and experienced a sharp economic decline due to the pandemic. Saudi Arabia, in particular, endured the lowest economic growth rate in nearly 35 years (58). The economic resilience of Greece ranked at the bottom among moderate resilience. The sudden onset of the pandemic caused Greece (0.021) to suffer a severe economic impact on its keystone national industries such as tourism and agricultural exports, and the unemployment rate rose to 19.9%. Russia (0.029) and Georgia (0.04) suffered from economic depression due to labor shortages caused by decreasing immigration. Most of the countries with low resilience are economically weak, such as Cambodia (0.07), Indonesia (0.06), and Myanmar (0.01), which have densely populated slums where the poor faced survival crisis during the quarantine. Other countries had week economic structures, such as Ukraine (0.029), Belarus (0.021), and Azerbaijan (0.014), which relied heavily on energy exports, with Azerbaijan’s oil and gas sector accounting for 45% of the economy, leading to severe economic setbacks during the pandemic (59).

The social resilience performance of countries with high and moderate resilience was relatively similar, with mean scores of 0.115 and 0.112, versus only 0.081 for the low-resilience countries. Israel (0.145), Cyprus (0.136), and Singapore (0.129) had the strongest social resilience among the high-resilience countries, with governments investing heavily in the health and education sectors to ensure adequate healthcare and high-quality literacy of their citizens. On the other hand, Qatar (0.093) performed poorly, with insufficient national education and still some way from achieving its goal of educational sustainability (60). In terms of social resilience, Slovenia (0.137) ranked first among the moderate-resilience countries, because of its general outstanding physical health and scientific literacy. Whereas India (0.086) has a low literacy rate, and the citizens generally lack scientific knowledge of pandemic prevention, making it difficult for the government to prevent pandemics. Pakistan had the worst performance (0.012) among the low-resilience countries, with a limited budget for higher education and a low expected years of education for its citizens, who have limited awareness of infectious risks and low compliance with social isolation policies (61).

The average technological resilience score of the high-resilience countries was 0.028, versus 0.024 for the moderate-resilience countries and 0.11 for the low-resilience countries. Israel (0.046), Singapore (0.040), China (0.035), and the UAE (0.033) had the strongest technological resilience. They had high rankings in the global innovation index and invested heavily in research and development, therefore enabled them to take the lead in developing vaccines (62). The Czech Republic had relatively strong technological resilience (0.035) and produces world-class medical equipment for export, whereas Vietnam (0.015) and India (0.014) had limited technological means to deal with a pandemic and need to learn from the experience of other countries. Countries with low resilience had a weak ground for scientific research and a low production capacity, which were far behind the countries with moderate and high resilience. As a result, they had to ask for vaccine assistance from other countries.

### 4.2. Policy recommendations for enhancing anti-pandemic resilience in B&R countries

Our study findings provide valuable policy recommendations to assist B&R countries in effectively responding to future pandemics. A rapid and proactive response from governments is crucial, particularly in the early stages of an outbreak when vaccines are unavailable and transmission mechanisms are unclear. To achieve this, it is essential to ensure the readiness of outbreak emergency preparedness structures and the establishment of an adaptive health system. This includes implementing non-pharmaceutical interventions like lockdowns and quarantine measures during the initial stages of an outbreak, buying crucial time for governments to enhance infection control measures, allocate medical resources, provide economic assistance, mobilize society, and accelerate the development of drugs and vaccines. Overall, a comprehensive multi-sector approach is required to ensure a holistic response to future health crises.

Second, the economic constraints created by policies designed to rigorously reduce the spread of the pandemic, such as lockdowns, are a key cause of inadequate resilience. Fearing that quarantine would cut off national income sources and lead to a severe economic crisis, many countries chose to avoid lockdowns and instead implemented a herd immunity strategy, which exacerbated spread of the virus (63). Some countries with high resilience combined strict defensive measures with economic relief programs during the early stages of the pandemic, and once vaccines became available, they began to balance disease prevention with economic development, with the goal of minimizing the cost of the fight.

Third, the pandemic was a stress test for each country’s infrastructure system. In some countries, limited infrastructure and uneven distribution created constraints on medical resources and energy, exacerbating the pandemic. Future infrastructure development should fully account for such extreme conditions to maximize the ability of infrastructure to combat a future pandemic. Furthermore, governments should not only focus on improving the healthcare system but should also develop the supply system by improving logistics and energy systems.

The COVID-19 pandemic has significantly impacted the physical and mental health of people globally, with vulnerable communities facing greater risk due to unequal access to economic, health, and education resources. In the long run, access to healthcare and education should be improved to enhance social resilience. Countries must prioritize improved pandemic preparedness that addresses the needs of vulnerable groups, including building trust and enhancing solidarity in COVID-19 responses, and creating a more equitable and resilient society (64).

Finally, we were reminded that “viruses have no border.” Governments must consider pandemics as major crises that create a common challenge for all humans. The B&R countries should make full use of this initiative to form a multilateral cooperation mechanism and build a community of human support for everyone. In addition, the B&R countries should share their experience in pandemic management and provide anti-pandemic assistance to countries that face greater challenges. Furthermore, scientific cooperation should be strengthened to develop effective drugs and vaccines as fast and as safely as possible. All countries should work together to provide global public goods that protect the health of their citizens and maximize the welfare of all human beings.

## 5. Conclusion

Based on a resilience perspective, we developed an indicator system to evaluate the anti-pandemic resilience of the B&R countries based on five dimensions: institutional, infrastructural, economic, social, and technological resilience. We used a three-scale method and an improved entropy method to assign weights to these dimensions, and found that the greatest weight was given to institutional resilience, followed by infrastructure, economic, and social resilience, with the least weight given to technological resilience. This resulted from our assumption that vaccines were not yet universally available in 2020, so it was most critical for governments to intervene to stop the spread of the virus. Infrastructure, economic, and social resilience were also important dimensions to contain the pandemic because they helped to mitigate the situation. In contrast, technological resilience reflected the development and production of vaccines, which played a limited role in the fight against the pandemic in 2020 and therefore carried less weight. This form of resilience became much more important in 2021, when vaccines became widely available, but that was outside our study period.

We used the VIKOR method to evaluate resilience, and used hierarchical clustering to classify countries into groups with low, moderate, and high resilience. We found 10 countries with high resilience. Of these, Israel, Bahrain, and China had the highest resilience due to a combination of institutional, social, economic, and technological advantages; 17 other countries, including Hungary, Vietnam, and Greece, had moderate resilience, with weak institutional and economic resilience; these countries faced the dilemma of how to balance economic development with control of the virus. Finally, we found 26 countries, including Myanmar, Laos, and Cambodia, with low resilience. These countries had inadequate infrastructure and other dimensions of resilience before the pandemic, so COVID-19 revealed and exacerbated long-term problems.

This study provides a comprehensive analysis of the anti-epidemic performance of Belt and Road countries and offers policy recommendations for dealing with future public health emergencies. Specifically, our study suggests that countries should enhance their epidemic prevention and control capacity, promote joint contribution and shared benefits in building resilient infrastructure, optimize the diversified economic pattern, strengthen the foundation of social resilience, and collaborate in the development of drugs and vaccines to provide public goods for the global community.

Our study has some limitations. We did not attempt to characterize all potentially relevant factors that lead to resilience. For example, rapid, accurate, and trustworthy communication with the public is essential for an effective pandemic response. Future research should examine ways to improve trust in governments through improved communication. In addition, We assigned equal weights to all second-level indicators of a given first-level indicator because we had no objective way to determine their relative importance, and did not account for the possibility that some of these indicators may be more important than others in certain countries. Furthermore, further research is needed to examine the impact of Belt the and Road Initiative on the epidemic resilience of countries along its routes. This will deepen our understanding of how the initiative strengthens countries’ ability to tackle future challenges. Additionally, our study did not include war-torn countries due to data limitations on their anti-pandemic systems. However, recognizing the unique challenges faced by these countries, we recommend conducting separate research to develop strategies for managing pandemics in such contexts. Despite these limitations, our study provides a strong framework for identifying factors that weaken resilience and mitigating these problems through government policies. With suitable modifications, our approach should be applicable elsewhere in the world.

## Data availability statement

The original contributions presented in the study are included in the article/Supplementary material, further inquiries can be directed to the corresponding author.

## Author contributions

LJZ, MMM, LXZ, and PLY were involved in literature search, study design, data collection, model building, result analysis, and writing. XYH and YQ were involved in review and editing. All authors contributed to the article and approved the submitted version.

## Footnotes

^1^ https://www.bsg.ox.ac.uk/research/research-projects/covid-19-government-response-tracker

^2^ https://www.dkv.global/covid-safety-assessment-200-regions

^3^ http://data.un.org/Default.aspx

^4^ https://data.worldbank.org/

^5^ https://www.who.int/data/gho

^6^ https://unctadstat.unctad.org/EN/

^7^ https://ourworldindata.org/human-development-index

^8^ https://www.wipo.int/ipstats/en/

^9^ https://www.worldometers.info/coronavirus/

^10^ https://www.ibm.com/analytics/spss-statistics-software

^11^ https://www.dkv.global/covid-19/full-report
