# Supplementary material for: Anti-pandemic resilience assessment for countries along the Belt and Road route
Source: Front Public Health. 2023 Nov 2;11:1152029. doi: 10.3389/fpubh.2023.1152029 (PMC10652767; doi:10.3389/fpubh.2023.1152029)
Supplement: Supplementary file 1 [file Table_1.DOCX]

# Supplemental Tables

Table S1 The definition of all abbreviations used in Figure 1 for the 53 countries included in the analysis.

| No. | Country name | Abbreviation | No. | Country | Abbreviation |
| --- | --- | --- | --- | --- | --- |
| 1 | Albania | ALB | 28 | Latvia | LVA |
| 2 | Azerbaijan | AZE | 29 | Lithuania | LTU |
| 3 | Bahrain | BHR | 30 | Malaysia | MYS |
| 4 | Bangladesh | BGD | 31 | Mongolia | MNG |
| 5 | Belarus | BLR | 32 | Myanmar | MMR |
| 6 | Bosnia and Herzegovina | BIH | 33 | Nepal | NPL |
| 7 | Brunei | BRN | 34 | Oman | OMN |
| 8 | Bulgaria | BGR | 35 | Pakistan | PAK |
| 9 | Cambodia | KHM | 36 | Philippines | PHL |
| 10 | China | CHN | 37 | Poland | POL |
| 11 | Croatia | HRV | 38 | Qatar | QAT |
| 12 | Cyprus | CYP | 39 | Republic of Moldova | MDA |
| 13 | Czech Republic | CZE | 40 | Romania | ROU |
| 14 | Egypt | EGY | 41 | Russian Federation | RUS |
| 15 | Estonia | EST | 42 | Saudi Arabia | SAU |
| 16 | Georgia | GEO | 43 | Serbia | SRB |
| 17 | Greece | GRC | 44 | Singapore | SGP |
| 18 | Hungary | HUN | 45 | Slovakia | SVK |
| 19 | India | IND | 46 | Slovenia | SVN |
| 20 | Indonesia | IDN | 47 | Sri Lanka | LKA |
| 21 | Iran | IRN | 48 | Thailand | THA |
| 22 | Israel | ISR | 49 | Turkey | TUR |
| 23 | Jordan | JOR | 50 | Ukraine | UKR |
| 24 | Kazakhstan | KAZ | 51 | United Arab Emirates | ARE |
| 25 | Kuwait | KWT | 52 | Uzbekistan | UZB |
| 26 | Kyrgyzstan | KGZ | 53 | Vietnam | VNM |
| 27 | Lao People's Democratic Republic | LAO |  |  |  |

| Table S2 Detailed descriptions of third-level indicators | |
| --- | --- |
| Third-level  Indicator | Description |
| Government lockdown policy stringency index | Data for the government lockdown policy stringency index was obtained from the Oxford Coronavirus Government Response Tracker (OxCGRT; <https://ourworldindata.org/covid-stringency-index>). The index is a composite measure of nine response metrics, including school closures, workplace closures, cancellation of public events, restrictions on public gatherings, closures of public transport, public information campaigns, restrictions on internal movements, international travel controls, and stay-at-home requirements. |
| Public health intervention policy index | Data for the public health intervention policy index was obtained from OxCGRT (<https://ourworldindata.org/grapher/covid-containment-and-health-index>). The index is obtained by calculating the average of four indicators: testing policy, the extent of contact tracing, requirements to wear face coverings, and policies around vaccine rollout. The result highlights the national government’s policy initiatives in epidemic prevention using health instruments. |
| Government efficiency of risk management index | Data for the government efficiency of risk management index was selected from the COVID-19 Regional Security Assessment Database (<https://www.dkv.global/covid-safety-assessment-200-regions>), which combines the performance of countries in six areas: level of security and defense advancement, rapid emergency mobilization, the efficiency of government structure, economic sustainability, pandemic readiness, and legislative efficiency. |
| Medical doctors (per 10,000) | Data for the number of medical doctors (per 10,000 people) came from the WHO Global Health Observatory database (<https://apps.who.int/gho/data/node.main.HWFGRP_0020?lang=en>), which counts the number of practicing physicians per million inhabitants and reflects the stock of medical human resources. |
| Hospital beds (per 1000 people) | Data for the number of hospital beds (per 1000 people) was obtained from the WHO Global Health Observatory database (<https://data.worldbank.org/indicator/SH.MED.BEDS.ZS>), which counts the number of hospital beds (for both acute and chronic care) per 1000 inhabitants, and reflects on aspects of the stock of medical supplies. |
| Nursing and midwifery personnel (per 10,000) | Data for the number of nursing and midwifery personnel (per 10,000 people) was obtained from the WHO Global Health Observatory database (<https://apps.who.int/gho/data/node.main.HWF1?lang=en>), which counts the number of practicing nursing and midwifery personnel per million inhabitants. |
| Current health expenditure as a percentage of GDP | Data for the current health expenditure as a percentage of GDP was obtained from the WHO Global Health Observatory database (<https://apps.who.int/gho/data/node.main.GHEDCHEGDPSHA2011?lang=en>), which measures the level of current health expenditure in monetary terms, and therefore reflects the importance of the health sector in the overall national economy. |
| Mortality rate attributable to unsafe water and inadequate sanitation and hygiene | Data for the mortality rate attributable to unsafe water, sanitation, and hygiene came from the WHO Global Health Observatory database (<https://apps.who.int/gho/data/node.main.SDG39?lang=en>), which counts the number of deaths per 100,000 people due to unsafe water and inadequate sanitation and hygiene. The indicator is used by international organizations to measure the effectiveness of countries in their fight against disease and death. |
| Percentage of the population using basic public health facilities | Data for the percentage of the population with access to the safe disposal of human urine and faeces was obtained from the WHO Global Health Observatory database (<https://apps.who.int/gho/data/node.main.SDG62?lang=en>), and was measured by counting the proportion of people who use basic health facility services (i.e., improved sanitation facilities that are not shared with other households). |
| Logistics performance index | Data for the logistics performance index was obtained from the World Bank database ([https://lpi.worldbank.org/international/aggregated- ranking?sort=desc&order=Infrastructure#datatable](https://lpi.worldbank.org/international/aggregated-ranking?sort=desc&order=Infrastructure#datatable)) and is measured using six dimensions: the quality of each country’s trade and transport infrastructure, the efficiency of customs, logistics development, level of connectivity, quality of service, and timeliness of delivery. |
| Access to electricity | Data for the access to electricity was obtained from the World Bank database (<https://data.worldbank.org/indicator/EG.ELC.ACCS.ZS>). It represents the percentage of the population with access to electricity and helps measure the extent of coverage of energy resources under the epidemic. |
| Electric power consumption | Data for the electricity power consumption indicator was obtained from the World Bank database(<https://data.worldbank.org/indicator/EG.USE.ELEC.KH.PC>). It measures the production of power plants and combined heat and power plants after subtracting the heat loss during transmission, distribution, transformation, and own use by heat and power plants. It therefore reflects the adequacy of the electricity supply in the event of an epidemic. |
| Percentage of individuals using the Internet | Data for the percentage of individuals using the Internet was obtained from the UN database (<http://data.un.org/Data.aspx?d=ITU&f=ind1Code%3aI99H>), which counts the share of a country’s population that has used the Internet in the past 3 months. |
| Mobile-cellular telephone subscriptions per 100 inhabitants | Data for the number of mobile cellular subscribers per 100 people was obtained from the UN database (<http://data.un.org/Data.aspx?d=ITU&f=ind1Code%3aI911>), which counts the number of mobile cellular subscribers using voice communications per 100 inhabitants in a country. |
| GDP growth rate in the last 5 years | Data for the GDP growth rate came from the World Bank database (<https://data.worldbank.org/indicator/NY.GDP.MKTP.KD.ZG>). The GDP growth rate for the last 5 years is calculated by averaging the GDP growth rate from 2015 to 2019, reflecting the growth rate of the country’s total economy and the overall level of economic strength in the last 5 years. |
| GDP per capita | Data on the GDP per capita was obtained from the World Bank database (<https://data.worldbank.org/indicator/NY.GDP.PCAP.CD>), using gross domestic product divided by the mid-year population, which is an important measure used to gauge the economic level of a country’s people. |
| The unemployment rate of the adult population | Data on unemployment rates for the adult population was obtained from the United Nations database (<https://data.worldbank.org/indicator/SL.UEM.TOTL.NE.ZS>), which counts the number of employed people who are not part of the labor force even though they are available for and seeking employment. |
| Life expectancy at birth | Data for the life expectancy at birth was obtained from the UN Human Development Report (<http://hdr.undp.org/en/content/latest-human-development-index-ranking>). This metric measures the average number of years a newborn is expected to live if mortality patterns at the time of its birth remain constant in the future and is a key indicator for assessing the health of a country’s population. |
| Universal healthcare (UHC) service coverage index | Data for the UHC service coverage index was obtained from the WHO database (<https://apps.who.int/gho/data/node.main.SDG38?lang=en>). The metric represents the percentage of the population that has access to the quality of health services they need (including medicines and other health products) without financial hardship. |
| Neonatal mortality rate | Data for neonatal mortality rates was obtained from the World Bank database (<https://data.worldbank.org/indicator/SH.DYN.NMRT>), and the indicator is the number of neonates who die before reaching 28 days of age, expressed per 1000 live births in a given year. |
| Literacy rate of the adult population | Data for the adult literacy rate was taken from the World Bank database (<https://data.worldbank.org/indicator/SE.ADT.LITR.ZS>). This metric represents the percentage of the national population aged 15 and older who can understand, read, and write short texts about their daily lives. |
| Expected years of schooling | Data on the expected years of schooling was obtained from the UN Human Development Report (<http://hdr.undp.org/en/content/latest-human-development-index-ranking>). This metric counts national calculations of the number of years children are expected to attend school and can be used as a yardstick for measuring the country’s educational development. |
| Government expenditure on education | Data on the government expenditure on education was obtained from the World Bank database (<https://data.worldbank.org/indicator/SE.XPD.TOTL.GD.ZS>). The data comprises public recurrent and capital expenditures on education as a share of GDP, and measures in monetary terms the importance that countries place on education. |
| Global innovation index | Data for the global innovation index was derived from the World Intellectual Property Organization database (<https://www.wipo.int/global_innovation_index/en/>) by averaging two sub-indices (innovation inputs and innovation outputs). The innovation input sub-index measures five major elements: institutions, human capital and research, infrastructure, market maturity, and business maturity. The innovation output measures two major elements: knowledge and technology outputs and creative outputs. |
| Research and development expenditure | Data on R&D expenditures was obtained from the World Bank database (<https://data.worldbank.org/indicator/GB.XPD.RSDV.GD.ZS>). The metric measures the share of current and capital expenditures (state and private), and is an internationally accepted standard for a country’s strength in science and technology. |
| Productive capacities index | Data for the productive capacities index was obtained from the UNCTAD database (<https://unctadstat.unctad.org/EN/Pci.html>). The overall index is based on eight factors: human capital, natural capital, energy, information and communication technologies, structural change, transport, institutions, and private sector trading. It reflects the state of a country’s capacity to produce goods and services. |

Table S3 Actual anti-epidemic resilience data for countries along the Belt and Road route in 2020. Countries are arranged from most to least resilient based on the data in Table 3.

| Country | Cumulative number of  confirmed diagnoses per million population | Cumulative number of  confirmed deaths per million population | Monitoring and detection score |
| --- | --- | --- | --- |
| Israel | 49283.3 | 391.2 | 143.1 |
| Bahrain | 58982.4 | 224.6 | 122.0 |
| China | 68.5 | 3.2 | 136.1 |
| Singapore | 10112.2 | 5.0 | 146.9 |
| United Arab Emirates | 21599.3 | 69.7 | 142.4 |
| Oman | 26650.2 | 309.9 | 114.6 |
| Qatar | 49924.0 | 90.9 | 123.4 |
| Saudi Arabia | 10806.4 | 185.2 | 120.5 |
| Kuwait | 35809.7 | 222.3 | 114.8 |
| Cyprus | 24796.0 | 100.1 | 126.8 |
| Hungary | 32980.5 | 973.2 | 119.9 |
| Estonia | 21418.9 | 175.2 | 95.4 |
| Vietnam | 15.1 | 0.4 | 106.0 |
| Greece | 12378.0 | 429.7 | 99.2 |
| Poland | 33628.6 | 735.3 | 94.2 |
| Slovenia | 57532.5 | 1280.5 | 91.2 |
| Turkey | 26187.8 | 252.0 | 115.2 |
| Lithuania | 52145.4 | 659.7 | 90.4 |
| Latvia | 20230.2 | 324.4 | 84.7 |
| Russia | 21752.2 | 385.6 | 77.7 |
| Georgia | 57815.6 | 635.0 | 96.7 |
| Czech Rep. | 66033.5 | 1075.6 | 97.5 |
| Brunei Darussalam | 350.2 | 6.9 | 84.6 |
| Malaysia | 3448.1 | 15.1 | 94.6 |
| Croatia | 50049.7 | 926.8 | 70.3 |
| Slovakia | 31786.0 | 378.9 | 87.3 |
| India | 7582.6 | 109.9 | 53.5 |
| Ukraine | 23752.9 | 416.4 | 74.5 |
| Romania | 32069.5 | 796.5 | 57.7 |
| Bulgaria | 28595.2 | 1067.9 | 75.4 |
| Albania | 19672.8 | 400.1 | 81.6 |
| Thailand | 93.1 | 0.9 | 79.3 |
| Azerbaijan | 21930.5 | 262.9 | 98.1 |
| Serbia | 49661.0 | 471.9 | 73.8 |
| Jordan | 29591.4 | 385.2 | 65.2 |
| Philippines | 4436.4 | 86.7 | 53.5 |
| Kazakhstan | 10715.2 | 122.9 | 67.4 |
| Mongolia | 376.4 | 12.2 | 62.6 |
| Belarus | 20351.1 | 149.6 | 76.2 |
| Indonesia | 2755.4 | 82.3 | 55.1 |
| Sri Lanka | 2038.3 | 9.5 | 45.6 |
| Moldova | 35594.8 | 732.5 | 74.9 |
| Pakistan | 2376.5 | 50.0 | 71.5 |
| Bangladesh | 3080.5 | 45.3 | 62.3 |
| Nepal | 8778.6 | 62.3 | 63.3 |
| Uzbekistan | 2381.0 | 19.0 | 64.2 |
| Egypt | 1375.0 | 76.2 | 62.0 |
| Bosnia and Herz. | 31677.8 | 1156.0 | 66.5 |
| Kyrgyzstan | 13212.9 | 220.9 | 67.9 |
| Iran | 14860.7 | 671.8 | 55.1 |
| Myanmar | 2297.6 | 49.5 | 62.1 |
| Lao PDR | 5.9 | 0.0 | 62.2 |
| Cambodia | 22.4 | 0.0 | 62.2 |

Table S4 The actual anti-epidemic resilience ranking ($U_{p}^{'}$) of the Belt and Road countries; low ranks and high $U_{p}^{'}$ represent high resilience.

| Rank | Country | $U_{p}^{'}$ | Rank | Country | $U_{p}^{'}$ |
| --- | --- | --- | --- | --- | --- |
| 1 | China | 1.00 | 28 | Myanmar | 0.419 |
| 2 | Singapore | 0.958 | 29 | Bangladesh | 0.418 |
| 3 | United Arab Emirates | 0.821 | 30 | Egypt | 0.416 |
| 4 | Saudi Arabia | 0.817 | 31 | Poland | 0.416 |
| 5 | Vietnam | 0.770 | 32 | Nepal | 0.405 |
| 6 | Cyprus | 0.735 | 33 | Kyrgyzstan | 0.402 |
| 7 | Oman | 0.677 | 34 | Bahrain | 0.379 |
| 8 | Turkey | 0.672 | 35 | Ukraine | 0.378 |
| 9 | Malaysia | 0.669 | 36 | Indonesia | 0.358 |
| 10 | Brunei | 0.604 | 37 | Philippines | 0.340 |
| 11 | Greece | 0.603 | 38 | Hungary | 0.329 |
| 12 | Azerbaijan | 0.597 | 39 | India | 0.326 |
| 13 | Estonia | 0.592 | 40 | Slovakia | 0.311 |
| 14 | Kuwait | 0.575 | 41 | Jordan | 0.300 |
| 15 | Thailand | 0.565 | 42 | Sri Lanka | 0.300 |
| 16 | Pakistan | 0.491 | 43 | Moldova | 0.293 |
| 17 | Israel | 0.482 | 44 | Serbia | 0.279 |
| 18 | Latvia | 0.482 | 45 | Lithuania | 0.241 |
| 19 | Albania | 0.451 | 46 | Iran | 0.221 |
| 20 | Belarus | 0.451 | 47 | Georgia | 0.208 |
| 21 | Qatar | 0.450 | 48 | Bulgaria | 0.190 |
| 22 | Uzbekistan | 0.440 | 49 | Croatia | 0.166 |
| 23 | Lao PDR | 0.435 | 50 | Romania | 0.160 |
| 24 | Cambodia | 0.435 | 51 | Bosnia and Herz. | 0.103 |
| 25 | Mongolia | 0.435 | 52 | Czech Rep. | 0.021 |
| 26 | Kazakhstan | 0.420 | 53 | Slovenia | 0 |
| 27 | Russia | 0.420 |  |  |  |

Table S5 Resilience scores for the 53 Belt and Road countries based on the values of the five first-level dimensions of anti-pandemic resilience. Countries are arranged from most to least resilient based on the data in Table 3.

| Country | Resilience | | | | |
| --- | --- | --- | --- | --- | --- |
|  | Institutional | Infrastructure | Economy | Society | Technology |
| Israel | 0.388 | 0.088 | 0.091 | 0.145 | 0.046 |
| Bahrain | 0.406 | 0.101 | 0.058 | 0.103 | 0.021 |
| China | 0.396 | 0.069 | 0.091 | 0.106 | 0.035 |
| Singapore | 0.309 | 0.108 | 0.103 | 0.129 | 0.040 |
| United Arab Emirates | 0.334 | 0.109 | 0.090 | 0.111 | 0.033 |
| Oman | 0.360 | 0.075 | 0.078 | 0.112 | 0.015 |
| Qatar | 0.343 | 0.102 | 0.085 | 0.093 | 0.026 |
| Saudi Arabia | 0.384 | 0.090 | 0.044 | 0.114 | 0.019 |
| Kuwait | 0.381 | 0.105 | 0.054 | 0.102 | 0.014 |
| Cyprus | 0.300 | 0.067 | 0.076 | 0.136 | 0.027 |
| Hungary | 0.268 | 0.082 | 0.061 | 0.115 | 0.029 |
| Estonia | 0.221 | 0.094 | 0.079 | 0.126 | 0.031 |
| Vietnam | 0.247 | 0.060 | 0.084 | 0.098 | 0.015 |
| Greece | 0.275 | 0.077 | 0.021 | 0.130 | 0.025 |
| Poland | 0.228 | 0.086 | 0.068 | 0.122 | 0.028 |
| Slovenia | 0.242 | 0.086 | 0.066 | 0.137 | 0.032 |
| Turkey | 0.245 | 0.061 | 0.060 | 0.105 | 0.019 |
| Lithuania | 0.217 | 0.085 | 0.066 | 0.110 | 0.025 |
| Latvia | 0.197 | 0.073 | 0.055 | 0.110 | 0.024 |
| Russia | 0.211 | 0.076 | 0.029 | 0.106 | 0.019 |
| Georgia | 0.220 | 0.055 | 0.040 | 0.093 | 0.016 |
| Czech Rep. | 0.195 | 0.096 | 0.065 | 0.121 | 0.035 |
| Brunei Darussalam | 0.150 | 0.071 | 0.051 | 0.112 | 0.017 |
| Malaysia | 0.196 | 0.074 | 0.061 | 0.103 | 0.023 |
| Croatia | 0.151 | 0.072 | 0.041 | 0.111 | 0.023 |
| Slovakia | 0.167 | 0.077 | 0.057 | 0.112 | 0.024 |
| India | 0.211 | 0.036 | 0.062 | 0.086 | 0.014 |
| Ukraine | 0.161 | 0.069 | 0.029 | 0.105 | 0.015 |
| Romania | 0.145 | 0.067 | 0.065 | 0.099 | 0.018 |
| Bulgaria | 0.116 | 0.074 | 0.051 | 0.098 | 0.021 |
| Albania | 0.156 | 0.048 | 0.038 | 0.105 | 0.011 |
| Thailand | 0.129 | 0.063 | 0.044 | 0.112 | 0.021 |
| Azerbaijan | 0.192 | 0.049 | 0.014 | 0.075 | 0.010 |
| Serbia | 0.137 | 0.069 | 0.046 | 0.097 | 0.021 |
| Jordan | 0.150 | 0.057 | 0.030 | 0.097 | 0.013 |
| Philippines | 0.167 | 0.044 | 0.061 | 0.072 | 0.010 |
| Kazakhstan | 0.154 | 0.074 | 0.047 | 0.094 | 0.010 |
| Mongolia | 0.128 | 0.041 | 0.046 | 0.098 | 0.013 |
| Belarus | 0.093 | 0.069 | 0.021 | 0.114 | 0.018 |
| Indonesia | 0.126 | 0.042 | 0.060 | 0.075 | 0.009 |
| Sri Lanka | 0.128 | 0.034 | 0.045 | 0.075 | 0.010 |
| Moldova | 0.107 | 0.051 | 0.036 | 0.094 | 0.014 |
| Pakistan | 0.148 | 0.014 | 0.057 | 0.012 | 0.003 |
| Bangladesh | 0.169 | 0.014 | 0.087 | 0.036 | 0.004 |
| Nepal | 0.162 | 0.020 | 0.064 | 0.061 | 0.005 |
| Uzbekistan | 0.083 | 0.045 | 0.071 | 0.097 | 0.005 |
| Egypt | 0.115 | 0.045 | 0.064 | 0.074 | 0.010 |
| Bosnia and Herz. | 0.079 | 0.057 | 0.035 | 0.097 | 0.014 |
| Kyrgyzstan | 0.110 | 0.043 | 0.037 | 0.102 | 0.005 |
| Iran | 0.091 | 0.050 | 0.022 | 0.099 | 0.014 |
| Myanmar | 0.151 | 0.010 | 0.048 | 0.035 | 0.000 |
| Lao PDR | 0.070 | 0.015 | 0.079 | 0.042 | 0.004 |
| Cambodia | 0.030 | 0.033 | 0.076 | 0.049 | 0.004 |
